# Voice Pathology Detection Using Modulation Spectrum-Optimized Metrics

**DOI:** 10.3389/fbioe.2016.00001

**Published:** 2016-01-20

**Authors:** Laureano Moro-Velázquez, Jorge Andrés Gómez-García, Juan Ignacio Godino-Llorente

**Affiliations:** ^1^Center for Biomedical Technology, Universidad Politécnica de Madrid, Madrid, Spain

**Keywords:** modulation spectrum, speech, dysphonia, cross-validation, EER

## Abstract

There exist many acoustic parameters employed for pathological assessment tasks, which have served as tools for clinicians to distinguish between normophonic and pathological voices. However, many of these parameters require an appropriate tuning in order to maximize its efficiency. In this work, a group of new and already proposed modulation spectrum (MS) metrics are optimized considering different time and frequency ranges pursuing the maximization of efficiency for the detection of pathological voices. The optimization of the metrics is performed simultaneously in two different voice databases in order to identify what tuning ranges produce a better generalization. The experiments were cross-validated so as to ensure the validity of the results. A third database is used to test the optimized metrics. In spite of some differences, results indicate that the behavior of the metrics in the optimization process follows similar tendencies for the tuning databases, confirming the generalization capabilities of the proposed MS metrics. In addition, the tuning process reveals which bands of the modulation spectra have relevant information for each metric, which has a physical interpretation respecting the phonatory system. Efficiency values up to 90.6% are obtained in one tuning database, while in the other, the maximum efficiency reaches 71.1%. Obtained results also evidence a separability between normophonic and pathological states using the proposed metrics, which can be exploited for voice pathology detection or assessment.

## 1 Introduction

Speech not only conveys linguistic but also a large amount of information about the speaker, such as sex, age, regional origin, health, etc. (Benzeghiba et al., 2007). This fact has motivated the design of automatic systems exploiting the traits embedded in speech, such as those that perform an automatic analysis of the patients’ vocal conditions. In this respect, the evaluation of voice quality is typically addressed by means of two different procedures: a subjective perceptual evaluation of the patient’s voice, where a score is assigned according to the judgment given by a listener; or by following an objective approach based on acoustic analysis quantifying certain aspects of the vocal acoustic signal (Barsties and De Bodt, 2014). Despite the popularity of the perceptual analysis, the objectivity as well as the non-invasiveness, cost efficiency, and easiness of use of the acoustical analysis make this approach more desirable in clinical scenarios. Moreover, the subjectivity of the perceptual analysis enhances the need of automatic systems to detect pathological voices or objective parameters to assess voice quality. In this study, several detectors of pathological voices are deployed using only one parameter each, extracted from Modulation Spectrum (MS). The main purpose is to tune a group of MS-based metrics to optimize their individual use in the pathological voice detection. The automatic detection is carried out using a simple Equal Error Rate (EER) scheme because the automatic detection itself is not an objective but a mean to optimize correctly the use of these proposed parameters. After the tuning, these parameters can be used in the future in a clinical study to assess their convenience for clinical use or as an ensemble of features for an automatic detector of pathological voices. Therefore, the use of acoustic analysis can provide objectivity to the voice assessment regarding perceptual analysis avoiding the subjectivity problems derived from the expertise of the rater or other cultural and physical facts (Bele, 2005).

Generally, perceptual and acoustic analyses are performed on sustained vowels rather than continuous speech, mainly because this acoustic material is expected to generate a simpler acoustic structure that might lead to consistent and reliable perceptual judgments of voice quality (Parsa and Jamieson, 2001). However, analysis of sustained vowels alone may not capture all salient characteristics of a patient’s voice, and therefore, it has been suggested to conduct perceptual and acoustic analyses on both sustained vowels and connected speech (Awan et al., 2010). Notwithstanding, several systems have performed successfully in automatic voice pathology detection (Boyanov and Hadjitodorov, 1997; Parsa and Jamieson, 2001; Godino-Llorente et al., 2006a; Saenz Lechon et al., 2006; Arias-Londoño et al., 2011b) or in evaluation of voice quality (Linder et al., 2008; Godino-Llorente et al., 2010) using sustained vowels solely.

There exist in literature many indices assessing voice perturbations, which might be classified according to the nature of the processing in the signal. Some of them characterize *amplitude perturbations* of the speech, such as shimmer, Amplitude Tremor Intensity Index (ATRI), Relative Average Perturbation (RAP), etc. Others characterize *frequency perturbations* of the signal with indices, such as jitter, Pitch Perturbation Quotient (PPQ), smoothed Pitch Perturbation Quotient (sPPQ), or Frequency Tremor Intensity Index (FTRI) among others. Similarly, other indices such as Harmonics to Noise ratio (HNR) or Glottal-to-Noise Excitation ratio (GNE) analyze the *noise* contained in the signal or consider the underlying *non-linearity* of the speech, such as entropy. An extended classification of parameters can be found in Moro-Velázquez et al. (2015). Although some of the indices described in literature have provided success in evaluating vocal quality, the suitability of many of them has not been studied deeply. Indeed, in some cases such as in the use of Mel-Frequency Cepstral Coefficients (MFCCs) (Rabiner and Juang, 1993), they have been considered due to their success in other speech applications but have been roughly translated to the voice assessment task. This approach might not be the most effective one in terms of performance since a proper tuning of the parameters describing the index can be decisive when facing situations, such as pathology detection or voice quality assessment. Some of the studies have demonstrated the usefulness of the tuning process to select optimal margins of work in voice pathology screening. In Godino-Llorente (2010), authors study the effect of varying the bandwidth of the Hilbert envelopes and the frequency shift on the computation of GNE (Michaelis et al., 1997) for the classification between normal and pathological voices using the a corpus of 226 voices from the MEEI database (Massachusetts Eye and Ear Infirmary, 1994).

In the last decade, some studies have used new types of parameterization based on measurements on MS. MS provides a visual representation of sound energy in acoustic and modulation frequency axes (Atlas and Shamma, 2003; Singh and Theunissen, 2003) supplying information about perturbations related with modulation of the frequencies present in the voice signal. Numerous acoustic applications use these spectra to extract features from which some examples can be found in Chu et al. (2011), Lim et al. (2011), Fan et al. (2012) and Bozkurt et al. (2014). Although there are few publications centered in the characterization of dysphonic voices using this technique from which Markaki and Stylianou (2009, 2011), Arias-Londoño et al. (2011a), Carbonell et al. (2015), Chittora et al. (2015), and Mekyska et al. (2015) are some examples, it can be stated that MS has not been studied deeply in the field of the detection of voice disorders and in most of the cases, the studies do not provide well defined parameters with a clear physical interpretation but transformations of MS, which are not easily interpretable, limiting their application in the clinical practice.

A subset of the MS metrics described on this paper were proposed by Moro-Velázquez et al. (2015) for the automatic assessment of Grade and Roughness following the GRBAS scale (Hirano, 1981). In spite of their good results, the study does not ensure if the metrics can be tuned using different frequency margins to optimize results. In this study, a tuning procedure for several of these MS metrics or parameters is performed taking into account different setups. These parameters are Modulation Spectrum Homogeneity (MSH), Cumulative Intersection Level (CIL), Ratio of points Above Linear Average (RALA), and Modulation Spectrum Percentiles (MSP). In addition and in order to ensure a robust procedure, the metrics are optimized using two different databases, selecting the setup producing the more generalist performance. Finally, the third database is used to test the optimized metrics using a Gaussian Mixture Model (GMM) machine learning scheme.

Therefore, the present paper proposes to select the optimal frequency margins and window size among others in order to find the best parameterization ranges for discriminating disordered from normophonic voices when using a set of MS parameters. For the decision-making process, the efficiency of a classification system based on EER operating point is employed to assess the performance of each one of the tested MS metrics. Experiments are accomplished in two distinct pathological voice databases to study the reliability of the tuning process in an inter-database scenario. Thus, the ranges that exhibit a similar behavior in both tuning databases and good performance will be selected as recommended for parameterization. The resulting metrics can be used separately in a clinical scenario to assess the quality of voice or in an automatic detector or pathological voices. In the first case, a clinical study is needed to validate the correct use of the parameters. In the second case, a study of the different classification strategies must be achieved. These two cases are out of the scope of this paper and must be taken into account for future work, but as an example, the third database is used to test the ensemble of the optimized metrics in a GMM scheme for the detection of normal and pathological voices.

This paper is organized as follows: section 2 develops the theoretical background referred to MS; section 3 explains the experimental setup in which methodology and databases are detailed; section 4 includes all the figures and tables of results. Lastly, section 5 expounds the conclusions and future work.

## 2 Theoretical Background

### 2.1 Modulation Spectrum

MS provides information about the energy at modulation frequencies that can be found in the carriers of a signal. It is a three-dimensional representation where abscissa usually represents modulation frequency and ordinate axis depicts acoustic frequency, applicate, and acoustic energy. This kind of representation allows the observation of different voice features simultaneously, such as the harmonic nature of the signal and the modulations present at fundamental frequency and its harmonics.

To obtain MS, the signal is filtered using a short-Time Fourier Transform (sTFT) filter bank whose output is used to detect amplitude and envelope. This outcome is finally analyzed using FFT (Schimmel et al., 2007), producing a matrix *E* where MS values at any point can be represented as *E*( *f_a_*, *f_m_*). The columns at *E* (fixed *f_m_*) are modulation frequency bands and rows (fixed *f_a_*) are acoustic frequency bands. Therefore, *a* can be interpreted as the number of the acoustic band and *m*, the number of the modulation band, while *f_a_* and *f_m_* are the central frequencies of the respective bands. Due to the fact that values *E*( *f_a_*, *f_m_*) have real and imaginary parts, the modulus of the spectrum is represented as |*E*|. Throughout this work, the MS has been calculated using the *modulation toolbox library* ver 2.1 (Atlas et al., 2010). Some different configurations can be used to obtain *E*, where the most significant degrees of freedom are the use of coherent or non-coherent modulation (Schimmel and Atlas, 2005), the number of acoustic and modulation bands, and acoustic and modulation frequency ranges.

**Figure 1:**
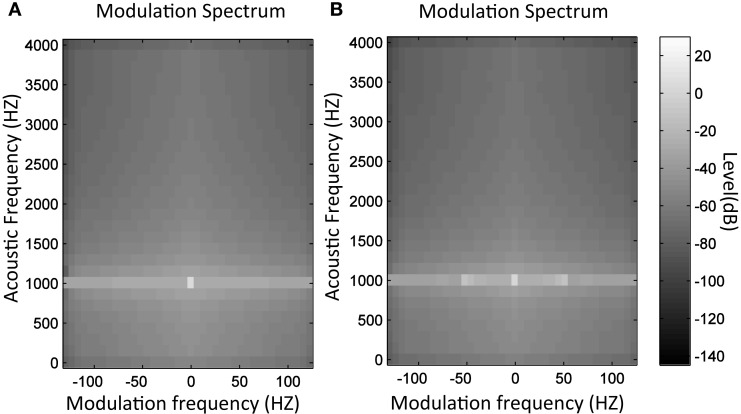
**MS modulus of 1 kHz sinusoidal tone (A) and 1 kHz tone with 50 Hz amplitude modulation (B)**.

**Figure 2:**
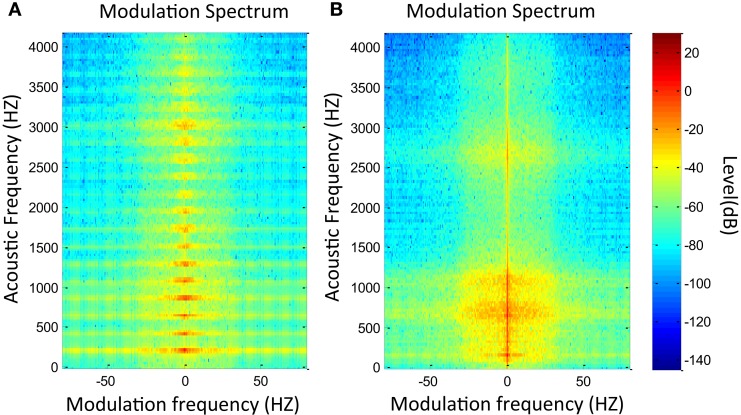
**MS modulus of a normal voice (A) and a pathological voice (B) with chronic hyperplastic laryngitis**.

Figure 1 illustrates an example of MS in which a sinusoidal tone is presented with and without amplitude modulation at 50 Hz. In the first image (Figure 1A), the tone is represented by a point at 1000 Hz in the acoustic frequency axis and at 0 Hz in the modulation frequency axis. In the second image (Figure 1B), the same tone is represented but including amplitude modulation at 50 Hz. In this case, two points emerge at 50 and −50 Hz in modulation frequency axis, while the central point remains but with lower level. Likewise, Figure 2 presents the MS of a normal voice (Figure 2A) and a pathological voice (Figure 2B). The harmonic character of the normal voice is easily observable unlike the pathological one. Moreover, in Figure 2A, most of the energy is located around the 0 Hz modulation band and in Figure 2B, there is more dispersion of energy.

One of the principal drawbacks of MS is that it provides a large amount of information, which has to be processed to obtain a more compact but precise enough representation of the speech segments. Thus, after obtaining the MS, some representative parameters are extracted to characterize voice quality. Some of the proposed metrics are described in Moro-Velázquez et al. (2015), such as the dispersion metrics (CIL and RALA) and modulus homogeneity (MSH). A new set of metrics is introduced, the MS percentiles which are used only at the present study and whose performance is compared with the former. All of these metrics use the MS modulus as input source and are briefly described next.

#### 2.1.1 Modulation Spectrum Homogeneity (MSH)

Representing MS modulus as two-dimensional images let observe that pathological voices usually have more complex distributions. Images related to normal voices are frequently more homogeneous and present less contrast. Accordingly, MS modulus homogeneity is used as a measurement of the existence of voice perturbations.

Homogeneity is computed using a variation of the Bhanu method described by Equation (1), as stated in Peters and Strickland (1990) utilizing *N* × *N* regions instead of 3 × 3 regions as proposed by Peters.

(1) $$\mathrm{MSH}=\sum_{a}\sum_{m}\left[E\left(f_{a},f_{m}\right)-{\bar{E\left(f_{a},f_{m}\right)}}_{N\times N}\right],$$

being *MSH* the MS Homogeneity value; *E*(*f_a_*, *f_m_*) the modulation spectra computation (modulus or phase) at point (*f_a_*, *f_m_*); and ${\bar{E\left(f_{a},f_{m}\right)}}_{N\times N}$ the average value in a *N* × *N* window centered at the same point.

#### 2.1.2 Cumulative Intersection Point (CIL) and Rate of Points above Linear Average (RALA)

As MS differs from normal to pathological voices, changes in the histograms of MS modulus reflect the effects of a dysfunction in a patient’s voice. A quick empirical overview of the MS permits to observe that pathological voices usually have a larger number of points with levels above the average value of |*E*|. The appearance of these points can be interpreted as the dispersion of the energy present in the central modulation band (0 Hz) toward side bands compared to the case of a normal voice.

**Figure 3:**
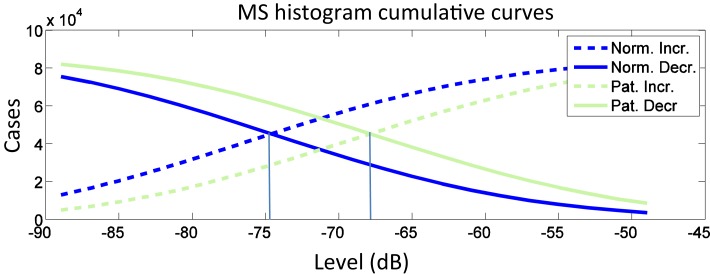
**Average cumulative curves for normal and pathological voices in MEEI subset**.

With this in mind, two metrics are proposed to measure such dispersion effect: Cumulative Intersection Level (CIL) and Ratio of Points Above Linear Average (RALA). CIL is the intersection between the histogram increasing and decreasing cumulative curves and matches the median. Histogram is processed from MS modulus in logarithmic units (dB). As shown in Figure 3, the CIL of the average cumulative curves of pathological voices on MEEI database is 7 dB higher than CIL of normal voices. On the other hand, RALA is the number of points in MS modulus, which are above average (linear units) divided by the number of points which are below this average.

(2) $$\mathrm{RALA}=\frac{\mathrm{NA}}{\mathrm{NB}}$$

being

(3) $$\mathrm{NA}=\sum_{f_{a}}\sum_{f_{m}}\gamma \left(f_{a},f_{m}\right)$$

(4) $$\mathrm{NB}=\sum_{f_{a}}\sum_{f_{m}}1-\gamma \left(f_{a},f_{m}\right)$$

and

(5) $$\gamma \left(f_{a},f_{m}\right)=\left\{\begin{array}{cc} 1\; & |E\left(f_{a},f_{m}\right)|\geq \bar{\left|E\right|}\\ 0\; & |E\left(f_{a},f_{m}\right)|<\bar{\left|E\right|} \end{array}\right.$$

where $\bar{\left|E\right|}$ is the MS modulus average, NA the number of points above $\bar{\left|E\right|}$, NB the number of points below $\bar{\left|E\right|}$, and NT the total number of points in *E*( *f_m_*, *f_a_*).

**Figure 4:**
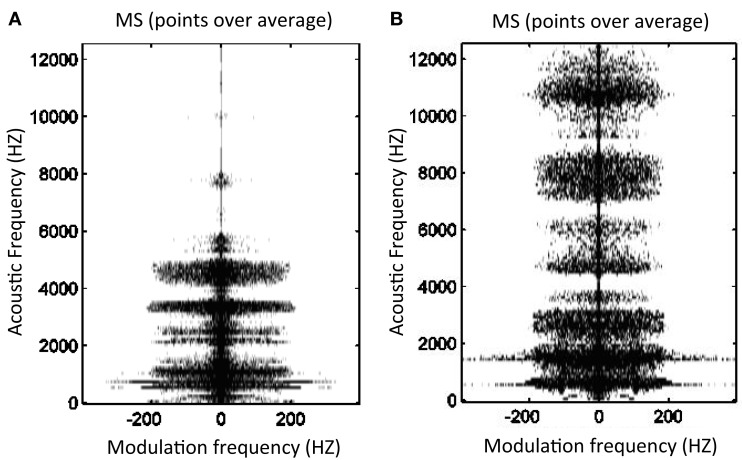
**Points above (black) and below (white) modulus average in MS for a normal voice (A) *RALA* = 0.12 and a pathological voice due to bilateral laryngeal tuberculosis (B) *RALA* = 0.27**.

Figure 4 represents these points in a healthy and a pathological voice. It is noticeable that, as expected, the MS of dysphonic voices present more points above the modulus average.

#### 2.1.3 Modulation Spectrum Percentile (MSP)

Taking into consideration MS modulus, 25, 75, and 95% percentiles are proposed as parameters. These statistical measurements, called MSP25, MSP75, and MSP95, respectively, point out the presence of energy on MS at different level ranges and serve as indicators of the level distribution at low, mid-low, and high relative level ranges given that all the signals into the database used to calculate these values are normalized. MSP95 indicates the value under which the lower level values are. MSP75 indicates the value in which the mid-low levels are around and MSP25 is referred to the mid-high levels. The higher the number of points of high levels in MS, the higher the MSP25 value. The higher the number of points of low levels, the lower the MSP95 value. By the way of illustration, with these metrics it is possible to compare if a voice has more high level or low level points at a particular modulation frequency range than other.

## 3 Experimental Setup

### 3.1 Databases

In this study, two widely used voice disorders databases are used to tune the proposed metrics, the MEEI database (Massachusetts Eye and Ear Infirmary, 1994) and the PdA database (Godino-Llorente et al., 2008). The purpose of using two databases is to perform a robust tuning and a comparison between the adjusting processes to ensure that the results are database-independent. Thus, the selection of the different frequency ranges and setups is performed in a more generalist manner. The third database, the HGM database, is utilized to test the optimized metrics using an advanced machine learning technique.

#### 3.1.1 MEEI Database

The MEEI voice database is used in this study. From the original 710 recordings of English speakers, a corpus of 226 including the sustained vowel /ah:/ is selected according to the criteria found in (Parsa and Jamieson, 2000). All the used files have been resampled to 25 kHz and 16 bits. Recordings of normal voices (53 files) have an average duration of 3 s, while pathological voices recordings (173 files) have an average duration of 1 s. The pathological voices include a variety of voice affections including organic, neurological, and traumatic etiologies. In Table 1, statistics from this database are depicted. This database has several drawbacks as it has been highlighted in Saenz Lechon et al. (2006), and its use could be controversial for obtaining absolute performance values in normal vs. pathological classification tasks. Its utilization in this study is aimed to validate the behavior of a tuning process with the PdA database and to serve as a comparison dataset for other studies due to its commercial availability.

**Table 1:** **Databases statistics**.

Database		Amount		Average age		Age range		SD	
		Men	Women	Men	Women	Men	Women	Men	Women
MEEI	Normal	21	32	38.8	34.2	26–59	22–52	8.5	7.9
	Pathological	70	103	41.7	37.6	26–58	21–51	9.4	8.2
PdA	Normal	85	112	28.7	31.0	18–64	13–66	12.9	13.1
	Pathological	61	114	44.7	34.8	19–68	16–65	11.7	12.4
HGM	Normal	41	54	37.6	40.0	17–78	19–85	15.4	16.0
	Pathological	32	78	49.1	47.2	26–80	18–81	15.6	16.2

#### 3.1.2 PdA Database

The PdA voice database is made up of Spanish speakers. An amount 372 voice recordings of the sustained vowel /ah:/ at 25 kHz and 16 bits are extracted from PdA database, excluding those recordings with high background noise or undetermined phonations. In Table 1, statistics from this database are depicted. The average duration of both pathological (175 files) and normal (197 files) voice recordings is 3 s. Fifteen pathologies are present in this database, mainly due to organic and traumatic etiologies.

#### 3.1.3 HGM Database

This third database is included to test the ensemble of all the metrics in a more complex machine learning scheme. The database was recorded in the hospital Gregorio Marañón of Madrid, and it includes voices of 107 patients and 95 control subjects. Although the database contains several types of recordings, for this study, only the sustained vowel /ah:/ has been selected, at sampling frequency of 22.05 kHz. In Table 1, statistics of age and sex are shown. The average duration of both pathological and normal voice recordings is 5 s. The voices were recorded using the microphone AKG *C*520 and the acquisition system Medivoz (Godino-Llorente et al., 2006b). HGM database includes 28 different causes of pathology including sulcus, edema, polyps, and paralysis among others. All the subjects recorded were assessed by a clinician previously, and in most of the cases, a laryngoscopy was performed.

### 3.2 Methodology

The main objective of this study is to select the most appropriate configuration to calculate a group of MS metrics for the detection of healthy vs pathological voices. The basic MS configuration corresponds to 1024 modulation bands, 128 acoustic bands, and Hilbert envelope in demodulation. Six measurements are accomplished in these spectra, MSH, CIL, RALA, MSP25, MSP75, and MSP95. The purpose is to identify the parameterization ranges by varying the different degrees of freedom that provides the best performance for pathology detection measured in terms of efficiency. Results are achieved using the described corpuses, MEEI and PdA separately in a k-folds validation technique, being k equal to 7. All the recordings from the three databases are conveniently normalized.

In the procedure, each metric is studied and tuned separately calculating its EER point for each group of training folds. This point is used to classify the sequences in the test fold between normal and pathological, and this performance is used to calculate the efficiency. This simple classification method has been chosen with the aim of observing the separability of normal and pathological voices for all the metrics. Taking into consideration that every single voice record is segmented in frames, all the frames coming from a certain speaker will be assigned to the same fold.

The main variables or degrees of freedom affecting the results of efficiency are frame length, mask size (*N* × *N*), upper modulation frequency limit, and acoustic frequency margins. Thus, four stages are defined at which one variable is varied into a range of values, while the rest of variables remain fixed. At every stage, both corpuses are parameterized several times taking into account the ranges of values and these producing the best efficiency are fixed for the next stage.

In the first stage, the frame length is varied in the range of 20–200 ms t 20 ms steps, while the other degrees of freedom remain fixed. Once the best frame length is identified, the size of the mask *N* × *N* is varied on a second stage, only affecting to MSH. After the selection of the more appropriate *N*, the third stage is defined in which the upper limit of modulation frequency is varied in the range of 20–220 Hz at 20 Hz steps to determine the importance of this bandwidth for the different metrics. Finally, a last stage is performed in which the three first variables are fixed with the selected values obtained in the previous stages. In that last phase, the influence of the acoustic frequency margins in efficiency is studied. The lower limit varies from 0 to 1000 Hz at 100 Hz steps and the upper between 1.2 and 12 kHz in 300 Hz steps at low frequencies and larger steps (from 1 to 3 kHz) at high frequencies. After efficiency calculation, optimal acoustic frequency ranges are obtained for each metric.

Lastly, the HGM database is parameterized two times to obtain the MS metrics. The first parameterization is accomplished using no restrictions, without following the optimization ranges and only in one frame length. The second one is accomplished using the ranges selected after the optimization process. The objective of these parameterizations is to observe the differences in efficiency using the selected ranges obtained after the optimization process respecting to the full range case when using the MS metrics as an ensemble in a GMM classifier. In order to obtain validated results, a k-folds scheme is utilized, where k is equal to 7. GMM tests are performed varying the number of Gaussians in the range of 4–64.

## 4 Results

**Figure 5:**
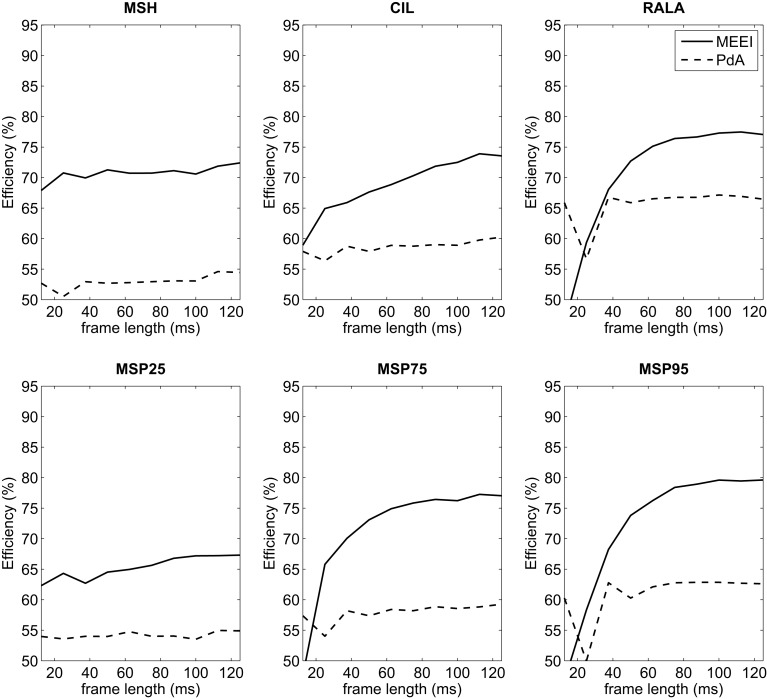
**Influence of frame length on efficiency for MEEI (continuous) and PdA (dashed) databases**.

In the first stage of tests in which frame length is varied, best results are obtained using 180–200 ms frames. All the tested metrics present their maximum efficiency in these frame lengths as it is deduced from Figure 5. Since the usage of 180 or 200 ms frames does not lead to absolute improvements over ±1% depending on the metric, 180 ms is selected as the basic frame length for the remaining tests in order to use a higher quantity of frames, and the shorter the frame, the better the assumption of stationarity.

**Figure 6:**
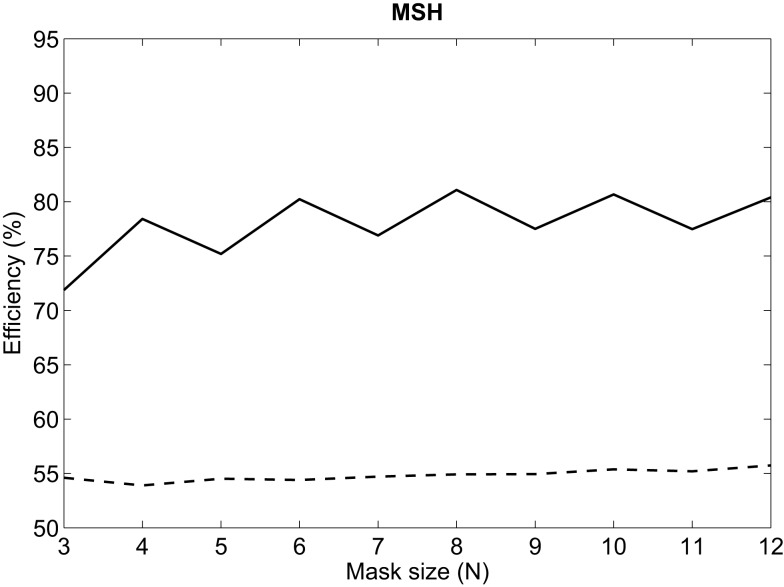
**Influence of the size of MSH mask on efficiency for MEEI (continuous) and PdA (dashed) databases**.

The influence of the mask size used to calculate MSH on efficiency is depicted in Figure 6. Best results are always obtained for even *N*. For even values larger than 6, results are quite similar, with an absolute variation of efficiency lower than ±1% in both tuning databases. Therefore, 6 × 6 is selected as the size of the mask used to calculate MSH.

**Figure 7:**
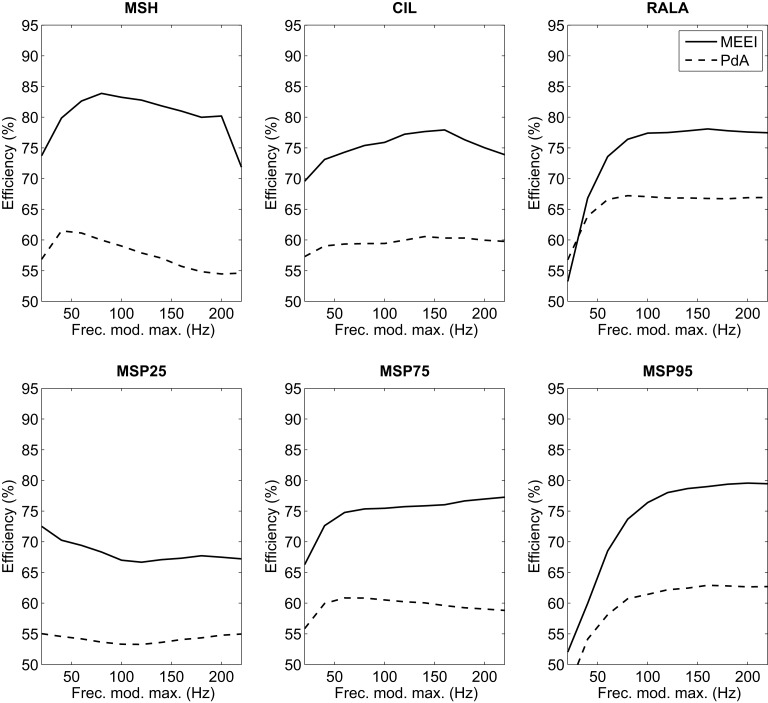
**Influence of high modulation frequency boundary on efficiency for MEEI (continuous) and PdA (dashed) databases**.

Hence, using 180 ms as frame length for all metrics and 6 × 6 masks (only applicable to MSH), a new round of efficiency calculations is accomplished. In this case, the upper limit of the modulation frequency is varied in the range 20–220 Hz with 20 Hz steps. Results are depicted in Figure 7. For MSH and MSP75, higher efficiency is obtained in the range of 50–100 Hz. In the case of CIL, this range goes from 120 to 160 Hz. RALA produces the best results over 80 Hz and MSP95 over 120 Hz. In the case of MSP25, best results are obtained employing 20 Hz as upper limit on modulation frequency axis. Thus, in the next stage, all tests are performed using 20, 80, 120, 140, and 200 Hz as upper limits of modulation frequency in MS for all parameters.

**Figure 8:**
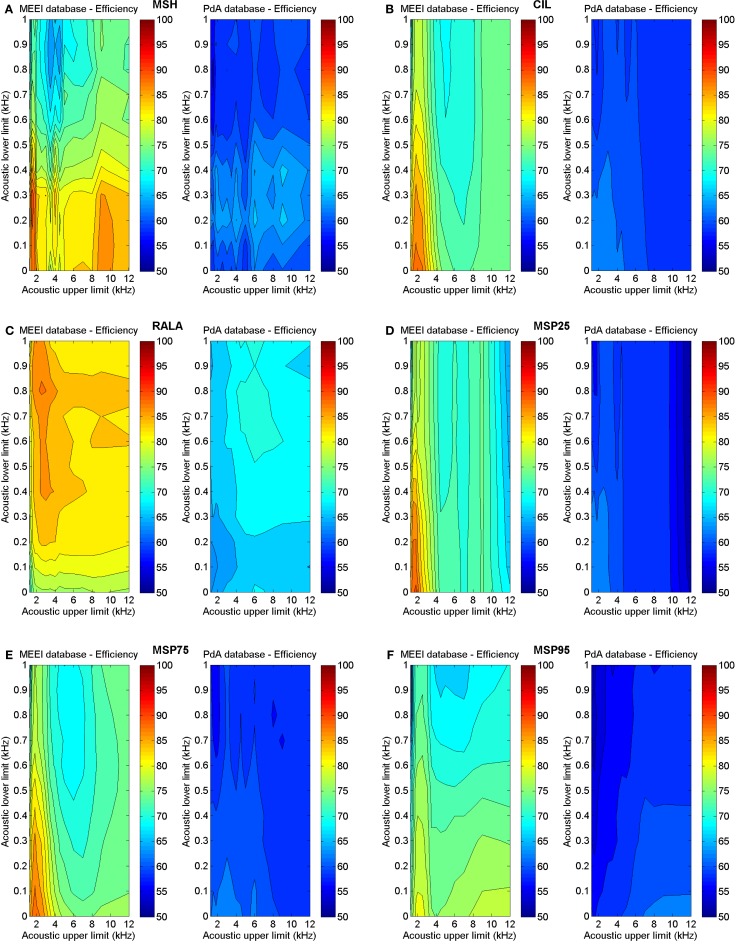
**Influence of acoustic frequency boundaries on efficiency in MEEI (left) and PdA (right) databases**. **(A)** MSH, **(B)** CIL, **(C)** RALA, **(D)** MSP25, **(E)** MSP75, and **(F)** MSP95.

Taking into account the results obtained in the previous stage, a new round of parameterizations is performed to calculate the variation of efficiency with lower and upper limits of acoustic frequency. Results are illustrated in Figure 8.

In Table 2, the ranges providing best results for each tuning database are displayed. As in all of the cases, the acoustic margins providing the best results overlap but do not match totally considering one tuning database with respect to the other. Thus, the acoustic margins are selected whose obtained results are reasonably good and are supposed to generalize in a better manner. These definitive acoustic ranges are included in Table 3.

**Table 2:** **Best efficiency results after tuning**.

MS metric	Database	Mod. freq. up. limit (Hz)	Acoustic range (Hz)	SD (%)	Best efficiency result (%)
MSH	MEEI	80	300–1500	±4.1	91.1
	PdA		400–6000	±7.1	66.7
CIL	MEEI	80	0–1800	±2.9	89.8
	PdA		0–3000	±5.4	63.9
RALA	MEEI	200	800–2500	±4.9	88.2
	PdA		800–6000	±8.2	71.1
MSP25	MEEI	80	0–1800	±4.1	90.6
	PdA		0–1800	±4.1	64.0
MSP75	MEEI	200	0–1800	±3.2	89.4
	PdA		0–3000	±6.5	63.8
MSP95	MEEI	200	0–2000	±4.0	80.9
	PdA		0–9000	±9.2	62.8

**Table 3:** **Efficiency results for the selected acoustic ranges**.

MS metric	Database	Mod. freq. up. limit (Hz)	Acoustic range (Hz)	SD (%)	Efficiency result (%)
MSH	MEEI	80	200–9000	±5.6	86.7
	PdA			±7.1	66.7
CIL	MEEI	80	0–2000	±4.0	89.3
	PdA			±4.0	63.5
RALA	MEEI	200	800–6000	±6.3	84.9
	PdA			±8.2	71.1
MSP25	MEEI	80	0–1800	±4.1	90.6
	PdA			±4.1	64.0
MSP75	MEEI	200	0–2000	±3.9	88.7
	PdA			±4.7	62.4
MSP95	MEEI	200	0–9000	±5.2	79.1
	PdA			±9.2	62.8

In Table 4, test results on HGM database are depicted. All calculations are made in 180 ms frames using a combination of the proposed metrics and a GMM classifier. In the optimized case, only the selected ranges proposed in Table 3 are used, while in the full range case, no restrictions are taken into consideration. Best results are obtained using the optimized metrics.

**Table 4:** **Efficiency results in HGM database using a GMM scheme**.

MS metrics	Efficiency% (confidence interval)
MS ensemble – full range	71.3 (6.2)
MS ensemble – optimized	72.3 (6.2)

**Figure 9:**
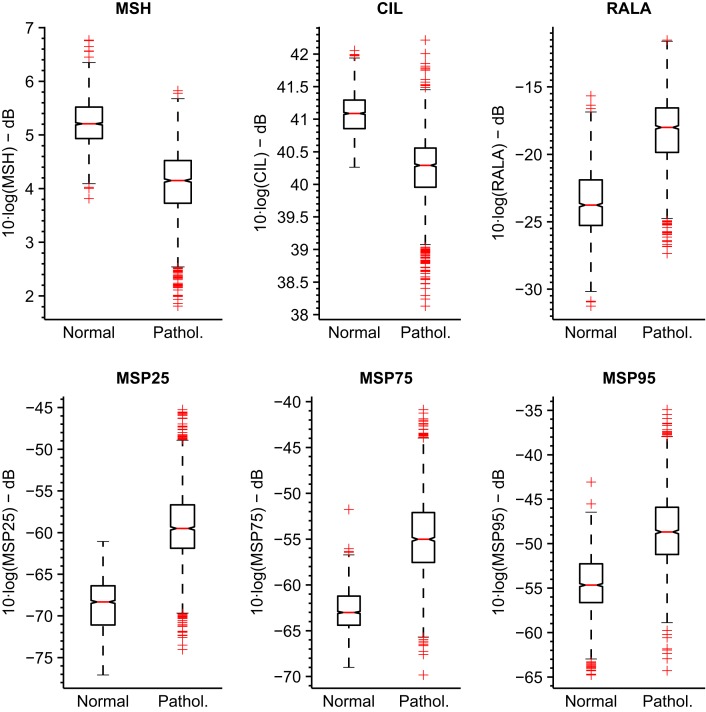
**Boxplots of tuned MS metrics for MEEI database**.

**Figure 10:**
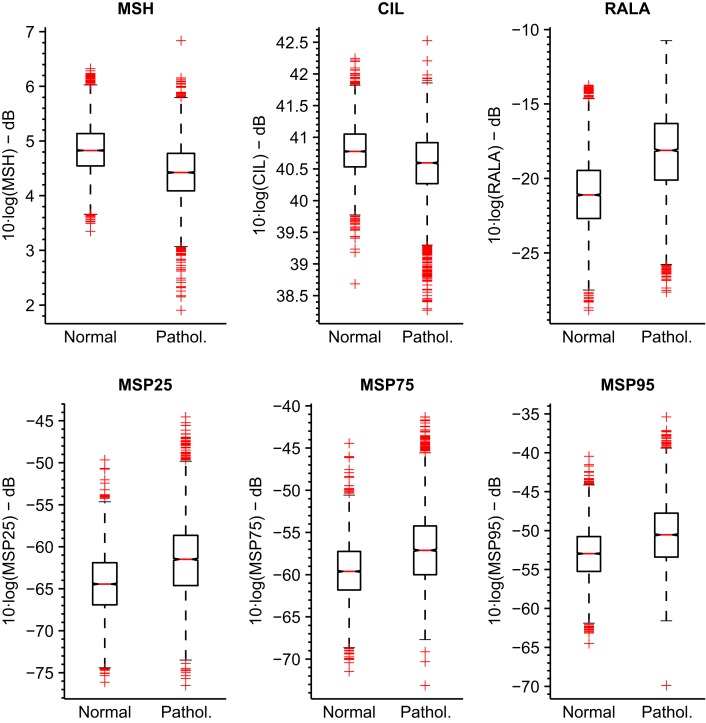
**Boxplots of tuned MS metrics for PdA database**.

The boxplots for these parameterizations are represented in Figure 9 for MEEI database and Figure 10 for PdA database.

Additionally, mutual information (Cover and Thomas, 2012) and Pearson’s correlation between all metrics have been calculated in order to analyze the relationship between them. Tables 5 and 6 incorporate the matrices of the relative mutual information for the selected ranges for MEEI and PdA, respectively. Tables 7 and 8 show the cross-correlation matrices for the selected ranges in MEEI and PdA databases, respectively. *P* values are always under 0.005.

**Table 5:** **Mutual information of metrics in MEEI database**.

MS metric	MSH	CIL	PALA	MSP25	MSP75	MSP95
MSH	0.99	0.33	0.18	0.03	0.04	0.04
CIL	0.33	0.89	0.16	0.05	0.05	0.05
PALA	0.18	0.16	0.68	0.02	0.02	0.05
MSP25	0.03	0.05	0.02	0.19	0.13	0.07
MSP75	0.04	0.05	0.02	0.13	0.19	0.08
MSP95	0.04	0.05	0.05	0.07	0.08	0.22

**Table 6:** **Mutual information of metrics in PdA database**.

MS metric	MSH	CIL	PALA	MSP25	MSP75	MSP95
MSH	1.00	0.17	0.07	0.01	0.01	0.02
CIL	0.17	0.98	0.01	0.01	0.02	0.02
PALA	0.07	0.01	0.54	0.00	0.00	0.01
MSP25	0.01	0.01	0.00	0.06	0.05	0.04
MSP75	0.01	0.02	0.00	0.05	0.10	0.04
MSP95	0.02	0.02	0.01	0.04	0.04	0.11

**Table 7:** **Cross-correlation matrix of metrics in MEEI database**.

MS metric	MSH	CIL	PALA	MSP25	MSP75	MSP95
MSH	1.00	0.82	0.81	0.80	0.79	0.79
CIL	0.82	1.00	0.72	0.92	0.92	0.82
PALA	0.81	0.72	1.00	0.71	0.69	0.75
MSP25	0.80	0.92	0.71	1.00	0.98	0.86
MSP75	0.79	0.92	0.69	0.98	1.00	0.90
MSP95	0.79	0.82	0.75	0.86	0.90	1.00

**Table 8:** **Cross-correlation matrix of metrics in PdA database**.

MS metric	MSH	CIL	PALA	MSP25	MSP75	MSP95
MSH	1.00	0.67	0.56	0.68	0.66	0.68
CIL	0.67	1.00	0.37	0.87	0.88	0.77
PALA	0.56	0.37	1.00	0.48	0.45	0.59
MSP25	0.68	0.87	0.48	1.00	0.94	0.82
MSP75	0.66	0.88	0.45	0.94	1.00	0.87
MSP95	0.68	0.77	0.59	0.82	0.87	1.00

## 5 Conclusion and Discussion

In this study, a tuning of six MS-based metrics is presented. Three metrics were introduced previously by the authors in Moro-Velázquez et al. (2015), while the remaining three are presented in the present paper, namely MSP25, MSP75, and MSP95. Concerning to the tuning process, frame length, mask size, modulation, and acoustic frequency ranges are varied to obtain maximum efficiency in the case of normal vs pathological detection using each parameter separately. Two tuning databases are used independently to first verify that the obtained results are validated and second to select variable values, which would produce a better generalization to detect pathological voices. Additional, the third database is used to test the optimized metrics in a GMM machine learning scheme.

Best results using the selected ranges are obtained with MSP25 in MEEI database in which 90.6% of efficiency is achieved and with RALA in PdA database, providing an efficiency of 71.1%. RALA seems to be the metric with better generalization in pathology detection, but it is important to notice that to find the best parameter is not the objective of this study but to optimize all of them. As each metric measures a different feature of MS, a combination of them should be used for classification purposes. It is difficult to claim what percentage of efficiency is the minimum required to use the parameters in a clinical stage. For instance, many controlled studies and tests made in laboratories obtained efficiencies close to 100% in speech recognition during the last decade, but that does not mean that the real-world automatic speech-recognition systems are infallible. The voice pathology detection case is quite similar. In order to validate a parameter for clinical use, a clinical study must be performed after the laboratory tests. The inclusion of new parameters in acoustic analysis software such as Praat (Boersma and Weenink, 2001), WPCVox, and MDVP (Godino-Llorente et al., 2008) facilitate the use of the new contributions and its integration in new clinical studies.

Regarding the first stage of the methodology, it can be deduced from Figure 5 that the tendencies in both tuning databases are quite similar in frame lengths larger than 80 ms. This means that for both databases, the performance increases as frame length increases and finally settles at 180 ms. This can be substantiated in the fact that most of the relevant information of MS is located at low modulation frequencies as it can be deduced from the third stage whose results are depicted in Figure 7. In all metrics but MSP25, performance decreases considerably if the upper limit of modulation frequency axis is restricted below 80 Hz. In these cases, poor results are obtained when the limit is 20 Hz. Accordingly, this supports the fact that the larger the frame length, the better the performance as short frames produce information leaks in very low frequencies. With reference to the second stage, which only affects MSH, results are better when using an even number for the mask length. This may be due to the differences between the modulus at a point, *E*( *f_a_*, *f_m_*), and the average level around it, ${\bar{E(f_{a},f_{m})}}_{N\times N}$, both values used in Equation (1). When *N* is odd, ${\bar{E(f_{a},f_{m})}}_{N\times N}$ is centered at ( *f_a_*, *f_m_*) and its value is more likely to be closer to *E*( *f_a_*, *f_m_*) than in the case in which *N* is even, when ${\bar{E(f_{a},f_{m})}}_{N\times N}$ is displaced from ( *f_a_*, *f_m_*). When using even *N* and having MS with low homogeneity, in most of the cases, pathological voices, MSH is more likely to be higher and the measurement of homogeneity is more effective as the results suggest.

Likewise, as it can be inferred from Figure 8, the behavior of the metrics is quite similar in both tuning databases concerning the acoustic frequency range with the distinction that efficiency is always higher using MEEI database. This aspect is important since it suggests a valid and database-independent behavior of the results. As for MSH, the most relevant acoustic range goes from 200 to 9000 Hz suggesting that the homogeneity around fundamental frequency does not seem to be important. Something similar happens with RALA whose lower limit is 800 Hz. In the rest of the cases, the acoustic lower limit is 0 Hz. For CIL, MSP25, and MSP75, the acoustic upper limit is around 2000 Hz, which means that the information present in the modulations of the upper harmonics is non-relevant with respect to these metrics. In the case of MSP95, the use of almost all the acoustic range produces the best results.

It is convenient to emphasize that there are some ranges in which the optimization process reveals different behaviors in the two databases. For instance, in Figure 5, it can be observed that efficiency around 40 ms frames rises up in PdA database for RALA, MSP75, and MSP95. In spite of the good results, 40 ms has not been selected as reference frame length for these parameters as its behavior is not reproduced in the MEEI database. Precisely, two databases have been used in this study to avoid selecting spurious behaviors, which could be corpus-adjusted. Similarly, it can be deduced from Figure 8 that the behavior of the tuning process in both databases is quite similar regarding the acoustic frequency limits although there are several differences. For instance, the efficiency of MSP95 (Figure 8F) in the MEEI database exhibits a crest of high efficiency under 3 kHz of acoustic upper limit, which does not appear in the PdA database. The behavior in MEEI database under 3 kHz can be considered as spurious and restricted to this database, while the crest over 8 kHz appears in both databases indicating that the selection of 9 kHz as acoustic upper limit is a more reasonable decision.

Regarding the correlation, Tables 7 and 8 show a strong correlation between parameters. This is not odd as all the metrics are done over the same matrix, MS, and are closely related one with regard to the other. For instance, CIL and MSPs are statistical percentiles of the same data and the tendencies in variation will be very similar for all of them. Mutual information is shown in Tables 5 and 6 from where it can be deduced that even after having a strong correlation, there exists separability between all the metrics. MSH, CIL, and RALA are less separable among them with respect to the MS percentiles and conversely.

It is noticeable that the MEEI efficiency results are always greater than the PdA results. Separability between normophonic and pathological voices is higher in the metrics calculated in the MEEI database as the boxplots in Figures 9 and 10 reflect. However, the literature has documented some issues regarding the MEEI database that might have biased the experiments (Saenz Lechon et al., 2006). In particular, it has been argued that the healthy and pathological patients were recorded at different locations and that some variability might have been introduced by means of this process. Nevertheless, the objective of this study is to determinate the different ranges of parameterization producing a good and corpus-independent performance instead of providing absolute efficiency values for the proposed metrics.

In Table 4, it can be observed that the use of the optimized parameters in a different database produces an improvement in performance of one global point respect to the non-optimized parameterization. In any case, for the future work, the tuned parameters may be used to detect pathological voices using several databases, different to those used in this study and comparing results with other typical parameters, such as perturbation or noise parameters. A study of the combination of all the parameters as the performed with the HGM database but comparing the achievement of several machine learning schemes will be of use. Moreover, its use in automatic classification of GRBAS or other perceptual assessment of voice is advisable. On the other hand, a more detailed study of the acoustic regions being selected for each parameter depending on the pathology is of interest due to it can reveal how physical impairments can influence the MS. In addition, a clinical study of the performance of these parameters in a clinical environment might be highly recommended. The tests in laboratory such as these presented in this study are the first step to the analysis of new metrics about voice pathologies, but the results obtained clinical studies define more clearly the usefulness of the parameterization procedures.

In conclusion, results suggest that the optimization of the proposed MS metrics for the detection of pathological voices increases efficiency. The tuning process ensures that the selected setup for calculating the metrics is not corpus-adjusted due to the similar evolution of the efficiency as a function of the setup variables in the two used databases.

## Conflict of Interest Statement

The authors declare that the research was conducted in the absence of any commercial or financial relationships that could be construed as a potential conflict of interest.
