# Corrigendum: Voice Pathology Detection Using Modulation Spectrum-Optimized Metrics

**DOI:** 10.3389/fbioe.2016.00067

**Published:** 2016-08-24

**Authors:** Laureano Moro-Velázquez, Jorge Andrés Gómez-García, Juan Ignacio Godino-Llorente

**Affiliations:** ^1^Center for Biomedical Technology, Universidad Politécnica de Madrid, Madrid, Spain

**Keywords:** modulation spectrum, speech, dysphonia, cross-validation, EER

**Reason for Corrigendum:**

There is a mistake in the values of the *x* axis of Figure 5 as published. The correct version of Figure 5 appears below. The article says that all tests have been performed in the range 20–200 ms but in Figure 5, *x* axis only includes the range 20–120 ms. The new figure includes the appropriate axis values. The authors apologize for the mistake. This error does not change the scientific conclusions of the article in any way.

**Figure 5:**
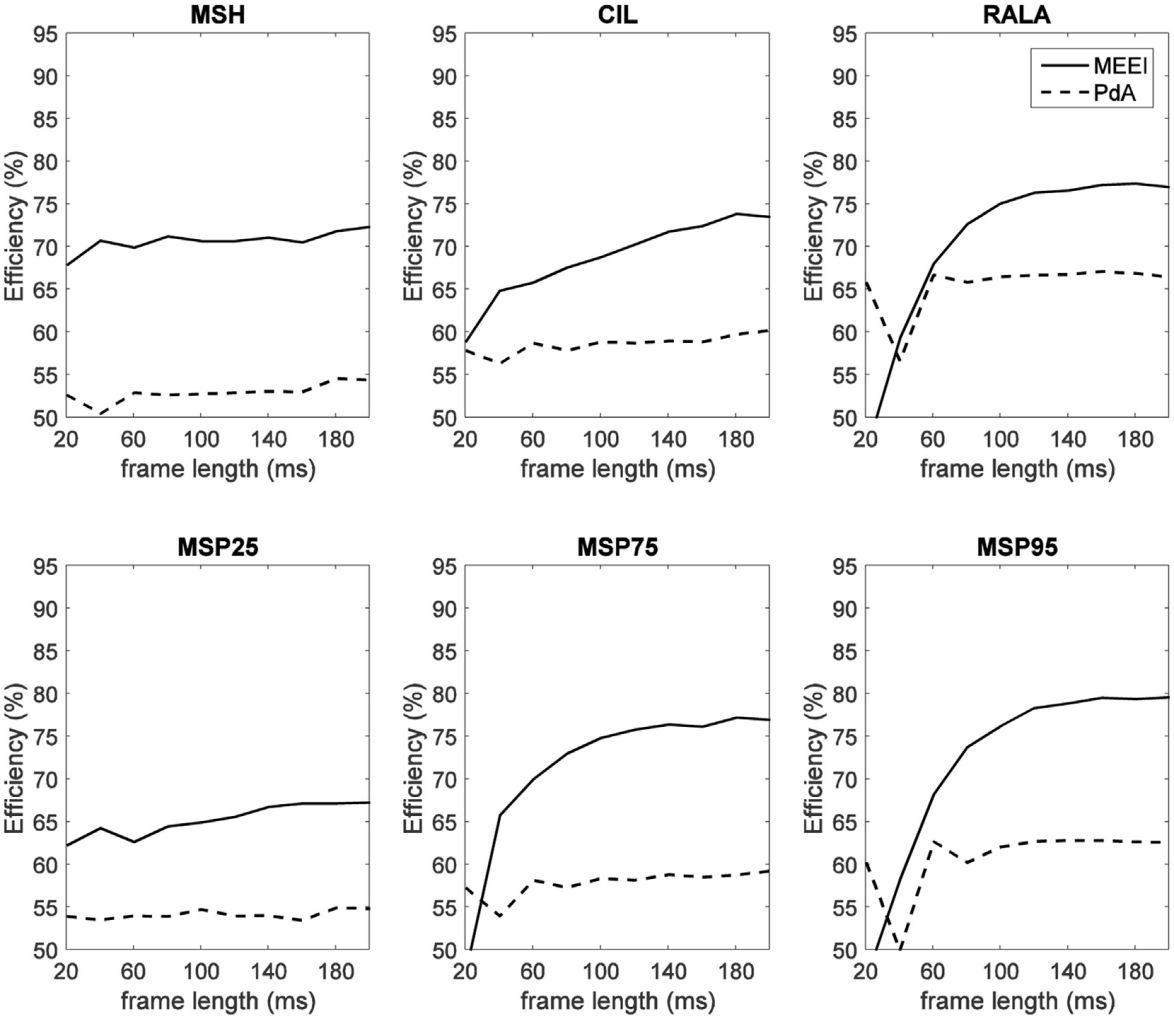
**Influence of frame length on efficiency for MEEI (continuous) and PdA (dashed) databases**.

## Conflict of Interest Statement

The authors declare that the research was conducted in the absence of any commercial or financial relationships that could be construed as a potential conflict of interest.

